# Study of Postacute Sequelae of COVID-19 Using Digital Wearables: Protocol for a Prospective Longitudinal Observational Study

**DOI:** 10.2196/57382

**Published:** 2024-08-16

**Authors:** Sherine El-Toukhy, Phillip Hegeman, Gabrielle Zuckerman, Anirban Roy Das, Nia Moses, James Troendle, Tiffany M Powell-Wiley

**Affiliations:** 1 Division of Intramural Research National Institute on Minority Health and Health Disparities National Institutes of Health Rockville, MD United States; 2 Biostrap USA, LLC Lakeway, TX United States; 3 Division of Intramural Research National Heart, Lung, and Blood Institute National Institutes of Health Bethesda, MD United States

**Keywords:** postacute sequelae of COVID-19, wearable devices, physiological parameters, prospective observational study, mobile phone

## Abstract

**Background:**

Postacute sequelae of COVID-19 (PASC) remain understudied in nonhospitalized patients. Digital wearables allow for a continuous collection of physiological parameters such as respiratory rate and oxygen saturation that have been predictive of disease trajectories in hospitalized patients.

**Objective:**

This protocol outlines the design and procedures of a prospective, longitudinal, observational study of PASC that aims to identify wearables-collected physiological parameters that are associated with PASC in patients with a positive diagnosis.

**Methods:**

This is a single-arm, prospective, observational study of a cohort of 550 patients, aged 18 to 65 years, male or female, who own a smartphone or a tablet that meets predetermined Bluetooth version and operating system requirements, speak English, and provide documentation of a positive COVID-19 test issued by a health care professional within 5 days before enrollment. The primary end point is long COVID-19, defined as ≥1 symptom at 3 weeks beyond the first symptom onset or positive diagnosis, whichever comes first. The secondary end point is chronic COVID-19, defined as ≥1 symptom at 12 weeks beyond the first symptom onset or positive diagnosis. Participants must be willing and able to consent to participate in the study and adhere to study procedures for 6 months.

**Results:**

The first patient was enrolled in October 2021. The estimated year for publishing the study results is 2025.

**Conclusions:**

This is a fully decentralized study investigating PASC using wearable devices to collect physiological parameters and patient-reported outcomes. The study will shed light on the duration and symptom manifestation of PASC in nonhospitalized patient subgroups and is an exemplar of the use of wearables as population-level monitoring health tools for communicable diseases.

**Trial Registration:**

ClinicalTrials.gov NCT04927442; https://clinicaltrials.gov/study/NCT04927442

**International Registered Report Identifier (IRRID):**

DERR1-10.2196/57382

## Introduction

Long-term health consequences have been identified as a core outcome domain for studies of patients with COVID-19 [1]. Dubbed as “long covid” [2] (or post–COVID-19 condition), postacute sequelae of COVID-19 (PASC) have been described on social media and in news media [3,4]. These reports referred to experiences of patients who take longer than normal time to recover or have more than 1 symptom that persists or develops after recovery [5,6]. This prompted calls to investigate the “prevalence, type, duration, and severity of persistent symptoms following resolution of acute SARS-CoV-2 infection, [and] risk factors associated with their development” [7]. Definitions of PASC varied depending on the onset and duration of symptoms, number and clustering of symptoms, patient population, and possibility of alternative explanations [8-11]. Heterogeneity in defining PASC translated into varying prevalence estimates, ranging from 43% to 80% in systematic reviews and meta-analyses of PASC among patients with COVID-19 at ~1+ month after infection [12-15]. Currently, the World Health Organization (WHO) defines PASC as a [16] “condition...in individuals with a history of probable or confirmed SARS CoV-2 infection, usually 3 months from the onset of COVID-19 with symptoms and that last for at least 2 months and cannot be explained by an alternative diagnosis.”

Patient-reported outcomes (PROs) and clinical evaluations show that patients with COVID-19 exhibit long-term health complications, regardless of initial disease severity [17-21], attributed to several hypothesized biological mechanisms [11,22,23]. These biological mechanisms include persistent viral reservoirs, sustained autoimmune and inflammatory responses, gut microbiome dysbiosis, endothelial dysfunction, and neurological signaling dysfunction [22,23]. PASC symptoms include fatigue, memory loss, and chest and abdominal pain, among 200+ other symptoms that affect multiple organs and systems [12,24-26], whereas emergent conditions include diabetes and hypertension [27,28]. Corroborating evidence shows persistent symptoms in recovered patients of other coronaviruses [29,30] and postviral chronic syndromes (eg, myalgic encephalomyelitis/chronic fatigue syndrome) [31]. Evidence of PASC is based on data primarily from hospitalized patients [18,19,32-35] and minimally from nonhospitalized patients [17,36], who represent ~20% and 80% of patients with COVID-19, respectively [37]. Longitudinal studies of PASC are limited because they have single-time outcome measurements [15], sparsely spaced data collection intervals, or short follow-ups (eg, [32,34,38,39]). Furthermore, studies focus on single symptoms or symptom clusters (eg, [40]), while few report psychological and well-being outcomes (eg, [18,33-35,41]). This raises the need for prospective longitudinal studies of nonhospitalized patients with COVID-19 with extended follow-ups to understand the nature, duration, and multisymptom manifestation of PASC [42,43].

Wearable devices continuously capture physiological parameters, which have been beneficial in detecting COVID-19 and monitoring its acute-phase symptoms [44-59]. These studies show that physiological, sleep, and activity parameters differ in the preinfection versus acute phases of the disease, symptomatic versus asymptomatic patients, and patients with COVID-19 versus healthy people. Physiological parameters collected via wearables parallel clinical indicators (eg, elevated respiratory rate) [60] and self-reported symptoms (eg, shortness of breath) [25] documented in patients and are associated with PASC [61,62]. Wearables-based studies of PASC are scarce and have short follow-ups [63,64]. COVID-19 wearables studies have several limitations including a focus on select parameters (eg, [54,64]); use of custom or specialized devices (eg, [59,65]), which limits their applicability and population-level impact; or recruitment of participants who already own wearable devices (eg, [50,54]), thereby introducing between-device variations in parameter calculations and self-selection biases [66].

This protocol outlines the design, objectives, and procedures of a 6-month decentralized prospective, observational study of PASC in a cohort of 550 nonhospitalized patients with a confirmed positive COVID-19 diagnosis.

## Methods

### Design

This is a single-arm, prospective, observational study of PASC in a cohort of 550 nonhospitalized patients with a positive COVID-19 diagnosis.

### Objective

The study aims to identify physiological parameters collected by wearable devices that are associated with PASC.

### End Points

The primary end point is long COVID-19, defined as 1 or more symptom at 3 weeks from date of the first symptom onset or positive diagnosis, whichever comes first. The secondary end point is chronic COVID-19, defined as 1 or more symptom at 12 weeks from symptom onset or positive diagnosis [6,9-11].

### Hypothesis

There is an association between physiological parameters collected by wearable devices and patient-reported long or chronic COVID-19.

### Population

A total of 550 patients will be recruited based on the demographic composition of the US population (Table 1) [67]. A prospective research volunteer (PRV) must meet all the inclusion criteria to be eligible for enrollment. The inclusion criteria are, first, male or female adults, aged 18 to 65 years. The pediatric population younger than 18 years of age is excluded because it differs from adults who are 18 years or older in infection rates, symptom manifestation, and outcomes [68]. The older than 65 years of age population is excluded because of its high-risk status, which prompted protections to reduce transmission among the older population, especially in nursing homes [69,70]. The second criterion is documentation of a SARS-CoV-19 polymerase chain reaction or antigen or rapid positive test, fewer than or 5 days before enrollment, issued by a public or private health care or COVID-19 testing facility, provider, or practitioner. The third criterion is ownership of or access to a smartphone or tablet, with an existing cellular data plan, that is compliant with the following specifications: (1) Android devices with an operating system of 6.0 or newer; Bluetooth 4.2, 5.x, or 6.0; and that implement Bluetooth Low Energy standard; (2) iPhone 6s or 6s plus (Apple) or newer; and (3) iPad mini 4 or newer, iPad 5th generation or newer, iPad Air 2 (second generation or newer), iPad Pro (9.7”, 10.5”, 11” first generation or newer), or 12.9-inch iPad Pro first generation or newer. The fourth criterion is English language proficiency. Non-English speakers will be unable to use the study mobile app, which is available only in English. The fifth criterion is the ability to understand and willingness to consent to participate. The sixth criterion is stated willingness to comply with all study procedures and availability for study duration. Finally, agreement to adhere to lifestyle considerations throughout the study (eg, wearing the wristband and temperature patch) is needed.

**Table 1:** Population by sex, race, and ethnicity in the United States, 2019 estimates for 18- to 64-year-old adults and study sample (N=550).

Ethnicity and race			US population, n (%)							Study sample, n (%)^c^				
			Male^a^		Female^a^		Total^b^		Male			Female		Total
**Non-Hispanic or Latino**														
	White	61,422,351 (50.1)		61,189,372 (49.9)		122,611,723 (73)		164 (50.2)			163 (49.8)		327 (73)	
	Black	13,264,328 (48.1)		14,324,175 (51.9)		27,588,503 (16.4)		36 (48.6)			38 (51.4)		74 (16.4)	
	American Indian and Alaska Native	1,311,132 (48.8)		1,376,957 (51.2)		2,688,089 (1.6)		3 (42.9)			4 (57.1)		7 (1.6)	
	Asian	6,788,614 (47.8)		7,418,266 (52.2)		14,206,880 (8.5)		18 (47.4)			20 (52.6)		38 (8.5)	
	Native Hawaiian and Pacific Islander	376,873 (50)		377,530 (50)		754,403 (0.4)		1 (50)			1 (50)		2 (0.4)	
	Total	83,163,298 (49.5)		84,686,300 (50.5)		167,849,598 (100)		222 (49.6)			226 (50.4)		448 (81.4)^d^	
**Hispanic or Latino**														
	White	17,271,789 (51.1)		16,500,664 (48.9)		33,772,453 (88)		46 (51.7)			43 (48.3)		89 (88)	
	Black	1,073,954 (48.9)		1,122,733 (51.1)		2,196,687 (5.7)		3 (50)			3 (50)		6 (5.7)	
	American Indian and Alaska Native	809,646 (52.1)		743,289 (47.9)		1,552,935 (4)		2 (50)			2 (50)		4 (4)	
	Asian	306,995 (49.7)		311,194 (50.3)		618,189 (1.6)		1 (50)			1 (50)		2 (1.6)	
	Native Hawaiian and Pacific Islander	116,272 (51.6)		108,954 (48.4)		225,226 (0.6)		1 (100)			N/A^e^		1 (0.6)	
	Total	19,578,656 (51)		18,786,834 (49)		38,365,490 (100)		53 (52.0)			49 (48.0)		102 (18.6)^d^	

^a^“Male” and “Female” cells for US population represent the percentage of each sex out of the total population by race or ethnicity (eg, percentage male individuals out of the total non-Hispanic or Latino White population).

^b^“Total” columns for US population represent the percentage of each race out of the total population by ethnicity (eg, percentage of White individuals out of the total non-Hispanic or Latino population).

^c^Cells for the study sample shown for each racial and ethnic category by sex based on US population aged 18 to 64 years for a sample of 550 patients.

^d^Cells represent total and percentage of the 550 target sample.

^e^N/A: not applicable.

An individual who meets any of the exclusion criteria is not eligible to enroll. The exclusion criteria are (1) residence outside of the US mainland, (2) enrollment in clinical trials on experimental COVID-19 therapeutics at baseline, (3) requirement of hospitalization at enrollment, (4) known history of allergic reaction to adhesives, and (5) inability to consent and unwillingness to comply with study procedures (eg, downloading a mobile app and sharing the data with the research team and providing required data such as close kin contact information).

### Recruitment

The Office of Patient Recruitment (OPR) created all recruitment materials and advertised the study in print and on the National Institutes of Health (NIH) Clinical Center television monitors and website [71]. Commercial marketing venues are used depending on recruitment pace and funding availability. Additionally, we partnered with Fulgent Genetics, a company that administers COVID-19 testing in Los Angeles, California. As part of their scheduling for COVID-19 testing, patients who express interest in participating in research studies are asked “would you be interested in sharing information you submitted during this registration process with researchers so they can contact you about studies?”

Only information about patients who have agreed to be contacted is accessed. Outreach activities are engaged in to increase the enrollment of minorities.

### Study Procedures

PRVs contact OPR’s recruitment center by phone or email. OPR’s staff screens interested PRVs for initial eligibility using yes or no questions. OPR staff shares basic demographic and contact information of eligible PRVs with the study team. PRVs who reach out directly to us or are part of Fulgent Genetics’ patient database receive an email and text invitation to enroll in the study, which includes a link to a proactive recruitment survey with yes or no questions like those that OPR uses to determine the initial PRV eligibility. These initial screening questions are about (1) PRV’s age; (2) having a confirmed positive COVID-19 test within the past 72 hours, which leaves us 2 additional days to enroll PRVs within the 5-day eligibility window; (3) having an Apple or Android smartphone or tablet with brand and model information; and (4) willingness to comply with study procedures. Initial screening occurs before patients provide an informed consent (IC) to participate in the study.

PRVs who pass the initial screening receive a welcome email inviting them to schedule an enrollment appointment. The email includes a link to an electronic IC, which states that this is a research study and outlines the schedule of activities (Table 2), risks and benefits to patients, and the voluntary and confidential nature of participation. The IC emphasizes that the study wearable devices should not be the basis of any medical decisions. During the appointment, we review the IC with patients who are given opportunities to ask questions and request time to consult with their families or physicians before consenting to participate. Patients who eConsent to participate undergo eligibility verification and are enrolled only if they meet all inclusion criteria and none of the exclusion criteria. Patients who have ≤4 GBs per person on their monthly cellular data plan and limited access to personal Wi-Fi are provided US $60 gift cards to purchase additional GBs from their phone carrier.

**Table 2:** Schedule of activities.

Procedures	Preenrollment	Enrollment, day 0	Day 1-30	Day 31-60	Day 61-90	Day 91-120	Day 121-150	Day 151-180
Screening	✓							
Informed consent		✓						
Eligibility verification		✓						
Baseline questionnaire		✓						
Physiological data collection (number of days)			30✓	8✓	8✓	8✓	8✓	8✓
Ecological momentary assessments (number of EMAs^a^)			30✓	4✓	4✓	4✓	4✓	4✓
Monthly survey (number of surveys)			1✓	1✓	1✓	1✓	1✓	1✓
Adverse outcomes survey (as applicable)			✓	✓	✓	✓	✓	✓
Technical or general support (patient-initiated)			✓	✓	✓	✓	✓	✓

^a^EMA: ecological momentary assessment.

Upon enrollment, patients are assigned a study ID and mobile app log-in credentials, which ensures patients do not use personal identifiable information to create an app account. Using the enrollment date, Research Electronic Data Capture (REDCap; Vanderbilt University) generates calendar dates that correspond to preloaded randomized data collection schedules that map the days on which each patient must perform study activities over 6 months (Table 3). The first day of device wearing starts on the third calendar day post enrollment to allow time for shipping an enrollment kit. During the first month, patients are required to wear a wristband and a temperature patch around the clock and answer a daily 2-question ecological momentary assessment (EMA). For months 2 to 6, patients are required to wear a wristband only for randomly selected 20 pairs of 2 consecutive days, resulting in an additional 40 device-wearing days in total. The schedule has a minimum of 48 hours buffer between 2 consecutive device-wearing periods, an approximately equal representation of weekdays and weekends where each day of the week appears 5 or 6 times during months 2 to 6, and an equal number of device-wearing days each month (ie, 8 days). For each 48-hour device-wearing period, patients answer a 2-question EMA. Patients are encouraged to wear the wristband and temperature patch upon receiving the enrollment kit, to wear the wristband beyond the 8 scheduled days per month during months 2 to 6, and to make up missed device-wearing days.

**Table 3:** Sample study schedule^a^.

Month and data collection days			Frequency and timing of study activities					
			Device wearing		EMAs^b^		Monthly survey (once)	
**Month 1 (daily)**								On April 2, 2023
	Days 1-30	From March 4, 2023, to April 2, 2023		From March 4, 2023, to April 2, 2023				
**Month 2 (4 times)**								On May 2, 2023
	Days 31 and 32	From wake up on April 4, 2023, to wake up on April 6, 2023		On April 6, 2023				
	Days 33 and 34	From wake up on April 10, 2023, to wake up on April 12, 2023		On April 12, 2023				
	Days 35 and 36	From wake up on April 19, 2023, to wake up on April 21, 2023		On April 21, 2023				
	Days 37 and 38	From wake up on April 28, 2023, to wake up on April 30, 2023		On April 30, 2023				
**Month 3 (4 times)**								On June 1, 2023
	Days 39 and 40	From wake up on May 8, 2023, to wake up on May 10, 2023		On May 10, 2023				
	Days 41 and 42	From wake up on May 16, 2023, to wake up on May 18, 2023		On May 18, 2023				
	Days 43 and 44	From wake up on May 20, 2023, to wake up on May 22, 2023		On May 22, 2023				
	Days 45 and 46	From wake up on May 28, 2023, to wake up on May 30, 2023		On May 30, 2023				
**Month 4 (4 times)**								On July 1, 2023
	Days 47 and 48	From wake up on June 4, 2023, to wake up on June 6, 2023		On June 6, 2023				
	Days 49 and 50	From wake up on June 9, 2023, to wake up on June 11, 2023		On June 11, 2023				
	Days 51 and 52	From wake up on June 14, 2023, to wake up on June 16, 2023		On June 16, 2023				
	Days 53 and 54	From wake up on June 28, 2023, to wake up on June 30, 2023		On June 30, 2023				
**Month 5 (4 times)**								On July 31, 2023
	Days 55 and 56	From wake up on July 6, 2023, to wake up on July 8, 2023		On July 8, 2023				
	Days 57 and 58	From wake up on July 10, 2023, to wake up on July 12, 2023		On July 12, 2023				
	Days 59 and 60	From wake up on July 20, 2023, to wake up on July 22, 2023		On July 22, 2023				
	Days 61 and 62	From wake up on July 25, 2023, to wake up on July 27, 2023		On July 27, 2023				
**Month 6 (4 times)**								On August 18, 2023
	Days 63 and 64	From wake up on August 4, 2023, to wake up on August 6, 2023		On August 6, 2023				
	Days 65 and 66	From wake up on August 12, 2023, to wake up on August 14, 2023		On August 14, 2023				
	Days 67 and 68	From wake up on August 19, 2023, to wake up on August 21, 2023		On August 21, 2023				
	Days 69 and 70	From wake up on August 24, 2023, to wake up on August 26, 2023		On August 26, 2023				

^a^Schedule is based on an enrollment date of March 1, 2023. Participation in the study is for 6 months with a total of 70 days of digital device wearing. In the first month, patients are required to wear a wristband and temperature patch, then only the wristband in months 2 to 6.

^b^EMA: ecological momentary assessment.

Patients are asked to complete 1 baseline and 6 monthly surveys. The baseline does not expire but patients are encouraged to complete it instantly after their enrollment appointment. Monthly surveys remain open for 12 days. Patients receive their data collection schedule, mobile app log-in credentials, and link to the baseline survey during their enrollment appointment. This allows us to verify that patients are receiving our electronic communications and to review these elements with them. We ship each patient an enrollment kit overnight, which includes (1) a print copy of their data collection schedule and their username for the mobile app account; (2) a wristband, charger, and sweatband; (3) a temperature patch, charger with batteries, and adhesives to secure the patch onto one’s body; (4) information sheet with first-time device set up instructions, data sync, and troubleshooting tips; (5) clinical trials informational sheet; and (6) a prepaid return slip with complete address information to ship back any defective devices.

For patients with no incoming wearables data and EMA responses for 3 consecutive days during the first month or 2 consecutive data collection periods during months 2 to 6, we attempt to contact patients to counsel them on the importance of adhering to their assigned study schedule. If we fail to reach a patient, we contact their close kin to complete an adverse events survey. Hospitalized patients are allowed to resume their participation in the study upon discharge and will complete an adverse events survey on their hospital stay. Upon completing the study, patients receive an end-of-study email thanking them and instructing them on how to create a personal mobile app account to continue using their wearable device should they choose to. After data analysis is complete, patients will receive a summary of the main findings.

### Study Evaluations

For 6 months, enrolled patients engage in various research activities. First, physiological parameters are passively collected through a wristband, Biostrap EVO Biometric set [72], and a Food and Drug Administration (FDA)-cleared temperature monitor, Vivalink [73]. The wristband is a noninvasive red and infrared optical sensor, which is less sensitive to skin tone variations and perfusion levels, and includes built-in accelerometer and gyroscope sensors that capture 6-axis motion data. Raw biosignals are used to estimate the (1) activity tracking accelerometer data captured continuously at 10 Hertz and converted into step count and activity duration saved in 1-minute increments; (2) arterial pulse volume monitoring using photoplethysmography, a 45-second recording collected at 43 Hertz, which provides derived biometrics including heart rate, heart rate variability with interbeat intervals between successive heartbeats times output, peripheral capillary oxygen saturation as percent oxygen, and respiratory rate; and (3) sleep analysis (eg, sleep duration, sleep onset and wake time, and sleep stages). The wristband technology has been validated previously [74]. The temperature patch collects body temperature captured continuously and reported as an average value each minute given the smartphone is within range. To ensure consistency in patient data, we set the sampling frequency to every 5 minutes and hid the settings option that allows patients to adjust the rate, duration, and intervals between measurements on the mobile app. Patients have instant and continuous access to their data. Second, EMAs capture COVID-19 symptoms and medications [75,76] that are collected in the mobile app (Table 4). Third, a baseline survey captures demographics and social determinants of health [75-77]; technology access [78,79]; COVID-19 symptoms and medications [75,76]; medical information [75,76]; risk and protective factors [78]; and PROs that capture cognitive, mental, and physical health domains [80-89]. Fourth, monthly surveys repeatedly capture data collected at baseline, mainly COVID-19 symptoms and medications [75,76], medical information [75,76], and PROs [80-89]. The first and last monthly surveys capture wearables acceptability data [90-92]. The last survey captures updated data on COVID-19 reinfection, vaccination, and medical care since enrollment in the study. Finally, the adverse outcomes survey captures the occurrence and timing of death or hospitalization, length of hospital stay, and treatment received [75,76].

**Table 4:** Study measures.

			Eligibility verification	Baseline survey		Monthly surveys													EMAs^a^			Adverse outcomes survey
						#1		#2		#3		#4		#5		#6						
Inclusion and exclusion questions			✓																			
Contact information			✓																			
Demographics			✓	✓																		
Social determinants of health				✓																		
**Technology access and acceptability**																						
	Smartphone or tablet access	✓		✓																		
	Internet or cellular data access	✓		✓																		
	Wearables acceptability				✓										✓							
**COVID-19 information**																						
	COVID-19 testing	✓													✓							
	COVID-19 vaccination	✓													✓							
	COVID-19 symptoms			✓	✓		✓		✓		✓		✓		✓		✓					
	COVID-19 medications			✓	✓		✓		✓		✓		✓		✓		✓					
**Medical information**																						
	Concomitant medications			✓																		
	Pregnancy and menstrual cycle			✓																		
	Disability			✓																		
	Existing and new health conditions			✓	✓		✓		✓		✓		✓		✓							
	Inpatient or outpatient care			✓	✓		✓		✓		✓		✓		✓					✓		
	Adverse events														✓					✓		
**Risk and protective factors**																						
	Body measurements			✓																		
	Fruit and vegetable intake			✓																		
	Physical activity			✓																		
	Tobacco use			✓																		
	Alcohol consumption			✓																		
	Marijuana use			✓																		
	Prescription and illicit drugs use			✓																		
**Patient-reported outcomes**																						
	General health			✓	✓		✓		✓		✓		✓		✓							
	Perceived health change			✓	✓		✓		✓		✓		✓		✓							
	EQ-5D-5L health status^b^			✓	✓		✓		✓		✓		✓		✓							
	EQ-VAS^c^																					
	PROMIS^d^-29 profile version 2.1 health-related quality of life^d^			✓	✓		✓		✓		✓		✓		✓							
	PROMIS cognitive function version 2.0 (short form 8a)			✓	✓		✓		✓		✓		✓		✓							
	PROMIS dyspnea severity version 1.0 (short form 10a)			✓	✓		✓		✓		✓		✓		✓							

^a^EMA: ecological momentary assessment.

^b^EQ-5D-5L: EurQol 5 domains, 5 levels. Captures 5 health dimensions: mobility, self-care, usual activities, pain or discomfort, and anxiety or depression.

^c^EQ-VAS: EurQol visual analog scale. Captures 7 domains: physical function, anxiety, depression, fatigue, sleep disturbance, ability to participate in social roles and activities, and pain interference, in addition to pain intensity.

^d^PROMIS: Patient-reported outcomes measurement information system.

Baseline, monthly, and adverse outcomes surveys are collected using REDCap electronic data capture tools hosted at the National Institute of Diabetes and Digestive and Kidney Diseases [93,94]. REDCap is a secure, web-based platform that serves as the project database and data capture tool. REDCap houses the IC and other study forms (eg, eligibility verification). The Biomedical Translational Research Information System REDCap team of the NIH Clinical Center provided REDCap project administration and consultation.

### Compliance and Retention

To maximize compliance with study activities, automatic reminders are sent to all patients. Additional reminders are sent to noncompliant patients. To maximize retention, we review an infographic aimed at increasing patients’ research literacy and highlighting the importance of completing research studies during their enrollment appointment [95]. Additionally, whenever there are 50 actively enrolled patients in a given month, protocol-compliant patients who completed their most recent monthly survey and scored ≥75% for device wearing and EMA completion are entered in monthly raffles for a US $100 gift card. Patients keep the wearable devices as a participation incentive.

### Statistical Analysis

We assume 20% to 35% event rates for the primary outcome of long COVID-19 and a 10% event rate for the secondary outcome of chronic COVID-19 [17,96].

### Power

A nonprobability sample of 550 patients with COVID-19 will be recruited for this study. With 550 participants, we have >85% power to detect a standardized difference (δ/σ) of 0.28, 0.33, and 0.43 in daily continuous physiologic parameters between long or chronic COVID-19 and non–long COVID-19 groups, assuming 35%, 20%, or 10% of participants will have long or chronic COVID-19, respectively, and allowing a 2-sided type I error of 5%. Our analysis will use multiple days of data per participant and will adjust for prognostic baseline factors, thus power will be substantially higher [97].

### Analysis Plan

We will use a linear mixed model of daily awake-time and sleep-time (calendar day) values of physiological parameters to determine associations with long and chronic COVID-19, the primary and secondary end points [98]. All valid days will be included for all subjects where a valid day consists of 18 hours of device wearing. Models will include baseline fixed-effect factors for demographic and clinical variables. A term for the COVID-19 status group (ie, non–long COVID-19, long COVID-19, and chronic COVID-19) will be included. All models will have a fixed-effect cubic model for time since testing positive or symptom onset for each COVID-19 status group. Subject-specific random effects (intercept and slope) and a spatial (in time) model will be used for within-subject errors, accounting for correlation between observations within a subject [98]. The cluster robust variance will be used [99,100]. The results of the above model will allow plotting differences between COVID-19 status groups at any time. For formal testing between groups, we will fit models exactly as described, but with only linear time models for each COVID-19 status group. Contrasts between the slope terms will then be used to test if groups have different trends over 6 months. Linear mixed-effect models are robust to missing data based on a missing-at-random assumption [98]. Subgroup analyses will be performed for analyses of both primary and secondary events restricted to patients within demographic characteristics (eg, race and ethnicity).

### Data Security

Data from wearables are transferred to iOS or Android mobile phones via Bluetooth and stored in Biostrap cloud servers for data processing to estimate physiological parameters. Patients’ personal identifiable information and protected health information are not shared with Biostrap. Data are downloaded weekly to the study server at the National Institute on Minority Health and Health Disparities and a final data transfer will occur at the end of the study.

### Ethical Considerations

The protocol was reviewed and approved by the National Institute on Minority Health and Health Disparities’s Scientific Review Committee and NIH’s Chief Scientific Officer on April 26, 2021. NIH’s institutional review board approved the protocol on June 21, 2021 (IRB#: 000315). Amendments were approved on September 23, 2021, and April 20, 2022. Administrative amendments were approved on June 17, 2022, August 24, 2022, May 16, 2023, June 30, 2023, and December 8, 2023 (ie, changes to the mailing address and key study personnel). Any adverse events or unanticipated problems are reported to the institutional review board. All patients enrolled in the study provide IC.

## Results

The first patient was enrolled on October 11, 2021. We expect to publish the results in 2025.

## Discussion

### Summary

This protocol is a prospective, longitudinal, observational study of PASC among a cohort of 550 nonhospitalized people with a confirmed positive COVID-19 diagnosis. The study captures intensive longitudinal physiological data collected via digital wearables and PROs for 6 months. The study will shed light on the course of PASC in nonhospitalized patients and represents an example of the use of wearable technology in disease monitoring generally and COVID-19 specifically.

Despite the pandemic emergency state declared officially over [101], research into PASC remains a priority given its health and economic costs [102,103], national initiatives [104,105], continued scientific interest [106,107], and patient advocacy [108-110]. Conservatively, there are 10 to 30 million people who experience PASC, with estimates likely higher if probable and suspected cases are included beyond the 100+ million confirmed COVID-19 cases in the United States [111]. The study will shed light on PASC in patient subgroups given its associations with demographics and risk profiles [34]. This is particularly important because evidence on PASC in racial and ethnic groups is scarce and inconclusive [11,17,40,61,112] despite minority groups disproportionally bearing the burden of COVID-19 infection and adverse outcomes [113]. While COVID-19 wearables-based studies focused mainly on its detection or acute phase, we capture a multitude of PASC digital biomarkers. This will allow us to identify PASC digital phenotypes that aid in its clinical assessment and treatment [114,115]. Furthermore, we can triangulate evidence of PASC from physiological parameters and self-reported patient outcomes [116]. Future studies should determine the clinical use of digital wearables in monitoring nonhospitalized patients with COVID-19 over longer follow-up periods.

Study strengths include its intensive longitudinal data on PROs and physiological parameters, 6-month follow-up, and focus on nonhospitalized patients whose characteristics and disease trajectory differ from hospitalized patients [13,117,118] (compare [12]). We require a positive COVID-19 diagnosis because of PASC-like self-reported symptoms and physiological changes among COVID-19 untested or negative individuals [26,41,46]. This is a decentralized trial, which allows for nationwide recruitment, increased participation of diverse and underrepresented populations in research, and reduced participant burden [119-121].

This study has several limitations. Sample size calculations are based on PASC surveillance data available when the study was launched. These data have since changed based on evolving definitions of PASC [16]. Recruitment started after COVID-19 vaccines were rolled out irrespective of age or underlying medical conditions, which means our sample will include unvaccinated and vaccinated patients. The availability and effectiveness of COVID-19 vaccines and treatments as well as dominant variants throughout recruitment activities may affect study end points [24,39,122,123]. Given limited evidence on the demographic breakdown of patients who endure PASC, our sample reflects the US population by sex and race and ethnicity rather than PASC prevalence in population subgroups [39]. The study mobile app is available only in English. Thus, we exclude non-English speaking patients from participation. Self-selection bias is another limitation whereby participants with certain predispositions (eg, digitally literate) may be inclined to enroll in the study. With a 5-day enrollment window from a positive COVID-19 diagnosis, the initial severity of one’s illness can deter PRVs from enrolling. We have no baseline data from patients prior to their positive diagnosis. Given the decentralized nature of the study, its reliance on mobile technologies, and its follow-up duration, certain limitations are expected (eg, data loss due to technical issues or shipping delays, poor compliance, or high attrition) [124-126]. The study wristband is not FDA-approved. Physiological parameters do not capture all PASC symptoms (eg, loss of sense of taste). Accrual is expected to fluctuate with infection rates, which may affect our ability to meet the target sample or prolong recruitment.

This study has wider public health implications pertaining to the role of mobile technologies in digital medicine [114,115,127,128]. Indeed, mobile technologies have been central to public health response to the COVID-19 pandemic [129,130]—from crowdsourcing platforms for contact tracing [131] to symptom reporting [132-134] and sensor data collection [107,135]. Wearables physiological data improve the identification of COVID-19–positive cases above and beyond self-reported symptoms [50], which have been the basis of pilot COVID-19 detection tools [46-48], and show preliminary differentiation between COVID-19 and other respiratory illnesses [136]. Prior to the COVID-19 pandemic, wearables have been piloted for the detection, monitoring, and public surveillance of communicable and noncommunicable diseases, health conditions, and behaviors [137-144]. They enjoy increasing uptake and acceptability, and afford opportunities for unobtrusive and continuous capture of objective data, including awake and sleep-time data, outside of traditional health care settings [114,145]. When integrated with remote monitoring platforms, patient-generated data can support real-time feedback to patients and physicians that is calibrated to a patient’s baseline data and has the potential to improve individual-level health outcomes [146,147] (compare [148]). While evidence on wearables and their integration into clinical practice is in its infancy and challenges exist until their potential is fully realized [147,149,150], if widely adopted, wearables can be scaled up as cost-effective tools for population-level disease surveillance, management, and prevention [150].

### Conclusions

This protocol outlines a 6-month prospective observational study of PASC in a cohort of 550 nonhospitalized patients with a positive COVID-19 diagnosis. The study uses off-the-shelf wearable devices to collect a multitude of physiological parameters to examine their associations with patient-reported PASC. The study exemplifies the potential of wearable technologies in population-level disease monitoring and their wider role in digital medicine.

## Appendix

Multimedia Appendix 1 Response to scientific review stipulations.

## Abbreviations

EMA: ecological momentary assessment
FDA: Food and Drug Administration
IC: informed consent
NIH: National Institutes of Health
OPR: Office of Patient Recruitment
PASC: postacute sequelae of COVID-19
PRO: patient-reported outcome
PRV: prospective research volunteer
REDCap: Research Electronic Data Capture
WHO: World Health Organization

## Data Availability

Data sharing is not applicable to this paper as no data sets were generated or analyzed during this study.

## Disclaimer

Use of commercial products in the study does not imply endorsement of these products. Similarly, authorship does not imply endorsement of any Biostrap USA, LLC or Vivalink products. Biostrap USA, LLC, was paid for the devices used in this study (Biostrap EVO Biometric set and Vivalink temperature monitor) and for the services it provided and has no input on study conceptualization, data analysis, or interpretation. The views expressed in this paper are those of the authors and do not necessarily represent the views of the National Institute on Minority Health and Health Disparities; the National Heart, Lung, and Blood Institute; the National Institutes of Health; or the US Department of Health and Human Services.
