# Supplementary material for: Study of Postacute Sequelae of COVID-19 Using Digital Wearables: Protocol for a Prospective Longitudinal Observational Study
Source: JMIR Res Protoc. 2024 Aug 16;13:e57382. doi: 10.2196/57382 (PMC11364950; doi:10.2196/57382)
Supplement: Multimedia Appendix 1 [file resprot_v13i1e57382_app1.docx]

**Response to Scientific Review Stipulations**

1. **Device and data quality.** Please provide information on the specific wristband that will be used to measure biometrics and any data to support the validity and reliability of biometrics collected with the specific device, especially among the targeted minority populations. Describe *specific* biometrics that will be collected via the wrist band.

**Response:** Information about the Biostrap device and biometrics collected appear in section 7.2 of the protocol. The Biostrap EVO wrist-worn device is a noninvasive optical sensor that monitors changes in arterial pulse volume using photoplethysmography (PPG). The device includes a built-in accelerometer and gyroscope sensors that capture 6-axis motion data for physical activity analysis, as well as removal of motion artifacts in the PPG data. The raw bio-signals recorded by the wristband device are transferred to the iOS or Android mobile phone via Bluetooth and stored in the Biostrap cloud servers for further data processing to estimate biometrics including:

- Heart Rate
- **
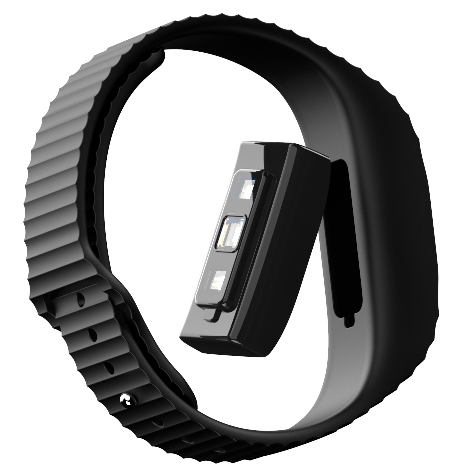
**Heart Rate Variability
- Oxygen Saturation
- Respiratory Rate
- Comprehensive Sleep Analysis
  - Sleep duration
  - Sleep onset and wake time
  - Sleep staging: awake, light & deep
  - Nocturnal biometrics
- Physical Activity
  - Hourly & daily step count
  - Active duration
  - Distance travelled

Please see the publication below for research on the validity and reliability of the biometrics obtained from the digital wristband. It is noteworthy that Biostrap uses red and infrared optical sensors, which are less sensitive to skin tone variations and perfusion levels because of their better penetration characteristics.

Dur, O., Rhoades, C., Ng, M. S., Elsayed, R., van Mourik, R., & Majmudar, M. D. (2018). Design rationale and performance evaluation of the wavelet health wristband: Benchtop validation of a wrist-worn physiological signal recorder. *JMIR mHealth and uHealth*, *6*(10), e11040.

1. **Representation of minorities**. Please clarify with 550 participants, how you will ensure representation of the targeted minority groups? How many are expected to have long covid-19? Chronic covid-19? How will you deal with potential decline in COVID-19 cases due to increasing vaccination rates? What subgroup analyses will be feasible and conducted?

**Response:** Table 3 in the protocol includes the sample breakdown by race and ethnicity. Section 5.7 in the protocol outlines strategies for recruitment. Specific to racial and ethnic minorities, we outline outreach efforts that include “circulating the study information on NIMHD’s website, listserv, and social media platforms; among PIs of cohort studies where minorities are over-represented (e.g., HANDLS) to pass it on to their participants per the protocols in place; among members of Community Advisory Boards and community liaisons with under-represented groups; and minority serving institutions such as Howard University and Eastern Virginia Medical School. Commercial marketing venues will be implemented depending on the recruitment pace and availability of funds).”

Research on long covid is in its infancy. Data on the racial/ethnic breakdown of patients who suffer from long covid are unknown.

The research team is aware of the increasing vaccination rates, which increases the difficulty of recruitment. However, we do not anticipate covid-19 infection rates to completely prohibit us from recruiting patients for the study. Factors such as vaccine supply and demand, vaccine hesitancy, and different covid19 variants contribute to infection rates currently at 60 thousand per day. In section 9.2, I outline circumstances under which the study will be discontinued and closed including the US reaching herd immunity. Section 8.2 outlines hazard ratios detectable at different sample sizes below the goal of 550 if we fail to reach our recruitment goals.

Section 8.3.4 of the protocol outlines the planned subgroup analysis by “(a) gender, (b) race/ethnicity (i.e., white vs. non-white), and (c) age group, which will be determined from the distribution of age in the study sample.” We aim to collapse data from racial/ethnic minorities vs. white in case our efforts to boost recruit minorities beyond the numbers reported in Table 3 fail.

1. **Monitoring for SAEs/AEs in the context of biometrics**. Please describe system to be used to alert participants to dangerous levels where a PCP should be contacted?

**Response:** The study will not alert participants to dangerous levels of any of the biometrics collected throughout the study. This decision is based on the following reasons:

1. The digital wristband device used in this study is not a medical grade device nor has it been approved by the Food and Drug Administration for such purpose. Accordingly, giving medical advice to patients based on physiological spikes potentially presents liability as it could be perceived as medical advice.
2. This is an observational study. Treating patients or intervening with their medical care is beyond the scope of the proposed research. In the consent form, patients are advised that they will not benefit from the study other than the potential benefit of having the ability to monitor their own biometrics.
3. The sampling of biometrics occurs every 5 minutes throughout the 24hour period of data collection days (i.e., first 30 days post enrollment, and 40 days for months 2 to 6). Setting up a system to monitor all recorded biometrics in real-time and alert patients if they reach a dangerous level requiring of medical intervention necessitates resources and expertise (e.g., physicians, computer scientists) that are currently not available for the research team. Traditionally, in published research where researchers have referred patients for medical care, this has been based on a single assessment of patients, typically done at baseline (e.g., screening for depression).
4. The threshold of “dangerous levels” such as heart rate is difficult to define as it would depend on various such as a patient’s baseline biometrics, age, sex, medical history, among others. Accordingly, the research team will not have sufficient information to determine the threshold that constitutes a dangerous deviation from baseline and deliver an alert to patients to contact their primary care physicians.
5. Finally, research is needed to determine whether an alert system should be based on a single biometric or a combination of biometrics; how would the alert system account for various factors such as patients’ age, baseline data, and medical history; whether the alert system would be based on a single deviation from the normal range or multiple deviant readings (and if so, how many) before an alert would be delivered to the patient; and finally, how would the alert system would avoid false positives based on erroneous readings from the digital wristband.
